# Chickpea diversity driven by transposon insertion polymorpism

**DOI:** 10.18699/vjgb-25-08

**Published:** 2025-02

**Authors:** V.A. Stanin, М.A. Duk, А.A. Kanapin, А.A. Samsonova, S.Yu. Surkova, М.G. Samsonova

**Affiliations:** Peter the Great St. Petersburg Polytechnic University, St. Petersburg, Russia; Peter the Great St. Petersburg Polytechnic University, St. Petersburg, Russia Ioffe Institute of the Russian Academy of Sciences, St. Petersburg, Russia; Peter the Great St. Petersburg Polytechnic University, St. Petersburg, Russia; Peter the Great St. Petersburg Polytechnic University, St. Petersburg, Russia; Peter the Great St. Petersburg Polytechnic University, St. Petersburg, Russia; Peter the Great St. Petersburg Polytechnic University, St. Petersburg, Russia

**Keywords:** chickpea, transposons, polymorphism, landraces, GWAS, adaptation, нут, транспозоны, полиморфизм, староместные сорта, GWAS, адаптация

## Abstract

Chickpea is the second most important legume crop, which is used as a food by people in different parts of the world due to its high nutritive value. Omics technologies have revolutionized the characterization of chickpea genetic diversity by considering single-nucleotide polymorphisms, while structural variants and transposons have been overlooked. The specific contribution of transposons to the phenotypic diversification of crop species is still poorly documented, therefore its characterization is important. We focused on landraces collected before the “green revolution”, as they are a valuable source of species diversity and can be used to broaden the genetic base of modern cultivars. Analyzing 190 chickpea genomes, we found 42,324 new transposon insertion sites from 83 families and showed that such sites are highly polymorphic. Most insertions were caused by mobilization of retrotransposons (67 % of insertions); among DNA transposons, the highest number of insertions was found for the superfamilies MuDR, PIF, hAT, CMC, and TcMar. We also demonstrated an uneven distribution of insertion sites along chromosomes. Analysis of the localization of transposon insertion sites relative to genes and their structural elements has shown that the largest number of insertions in all transposon superfamilies falls on introns and the smallest, on exons. We also showed that transposon insertion sites, which until recently have been overlooked by population genomics, are an important factor that diversifies phenotypes and can be used in GWAS as markers replacing SNPs. Comparative analysis of landraces collected in different geographic regions showed that the Ethiopian accessions have many unique transposon insertion sites. Our results highlight the unique role of transposon mobilization in chickpea diversification and have important implications for breeding improved chickpea varieties adapted to global climate change.

## Abstract (ru)

Нут – важная зернобобовая культура, которая используется народонаселением разных частей света в пищу в силу высокой ценности. Применение омиксных технологий позволило oхарактеризовать генетическое разнообразие нута, обусловленное однонуклеотидными полиморфизмами, тогда как структурные варианты и инсерции транспозонов выпали из поля зрения исследователей. Поэтому характеристика состава мобилома индивидуальных сортов нута и оценка его влияния на фенотипическую изменчивость и адаптацию актуальны. В фокусе нашего внимания были староместные сорта, собранные до «зеленой революции», поскольку они являются ценным источником видового разнообразия и могут быть использованы для расширения генетической базы современных сортов. Проанализировав 190 геномов нута, мы обнаружили 42 324 сайта инсерции транспозонов 83 семейств. Большинство инсерций (67 %) вызваны мобилизацией ретротранспозонов. Из ДНК-транспозонов наибольшее число инсерций найдено для суперсемейств MuDR, PIF, hAT, CMC и TcMar. Продемонстрирована неравномерность распределения сайтов инсерции вдоль хромосом. Анализ локализации сайтов инсерции транспозонов относительно генов показал, что наибольшее количество вставок у всех суперсемейств транспозонов приходится на интроны, наименьшее – на экзоны. Мы также показали, что сайты встройки транспозонов, которые до недавнего времени находились вне поля зрения популяционной геномики, являются важным фактором, диверсифицирующим фенотипы, что позволяет использовать их в полногеномном поиске ассоциаций в качестве маркеров наряду с однонуклеотидными полиморфизмами. Сравнительный анализ мобиломов сортов из разных географических регионов выявил существенное отличие эфиопских образцов от образцов других групп, собранных в Индии, Узбекистане, Турции, Средиземноморье, на юге России и в Ливане. Совокупность полученных нами данных и результатов – ценный ресурс, который может быть использован в качестве отправной точки для селекции улучшенных сортов нута, адаптированных к различным климатическим условиям.

## Introduction

Chickpea is one of the most important food legumes grown in many parts of the world, including Asia, Africa, North and South America and Europe. It accounts for 15 % of the world’s legume yield (Abbo et al., 2003; Jain et al., 2013). Chickpea is an important component of the diet for millions of people all over the world, providing protein, dietary fiber, unsaturated fatty acids, vitamins, macro and micronutrients

Chickpea is grown mainly in arid and semi-arid regions on poor soils (de la Peña, Pueyo, 2012). In these regions, various abiotic stresses such as water scarcity, extreme temperatures, short growing season affect chickpea productivity. For example, drought reduces global chickpea yields by 50 % and losses due to temperature extremes go up to 20 % (Kaloki et al., 2019). In such a scenario, identification and/or development of high-yielding genotypes is critical. These new chickpea varieties need to be resilient to climate change and adapted to changing consumer demands, agricultural practices and a wider climatic range. However, current elite chickpea varieties have low genetic diversity and do not contain useful alleles associated with tolerance to biotic and abiotic stresses. Hence, a broader genetic base is required for continuous production of new varieties

More primitive landraces collected before the “green revolution” are a valuable source of crop species diversity. Their use in plant breeding can lead to the development of resistant varieties with stable characteristics under unfavorable conditions. In the early 20th century, N.I. Vavilov systematically collected chickpea landraces, which are now stored at the N.I. Vavilov All-Russian Institute of Plant Genetic Resources (VIR) in St. Petersburg, Russia. This collection has been explored earlier to identify associations between SNPs and phenotypic traits using a single-locus genome-wide association study (Sokolkova et al., 2020).

Although the application of omics technologies has enabled large-scale characterization of germplasm, our understanding of the mechanisms underlying chickpea diversity is still limited. This situation is partly explained by the fact that until recently, for technical reasons, such studies have focused on the functional role of single nucleotide polymorphisms and short insertions/deletions (Varshney et al., 2019), while larger structural variants can account for a significant proportion of interspecific differences in DNA sequences. Most structural variants arise from the mobilization of transposons. Transposons constitute a significant part of the plant genome (Quesneville, 2020; Mhiri et al., 2022), and their movement leads to genome rearrangement, epigenetic silencing, and rewiring of gene networks (Bourque et al., 2018). Moreover, transposons are not randomly distributed in the genome and can serve as material for the emergence of new protein-coding genes and non-coding RNAs (Pulido, Casacuberta, 2023).

Transposons are a highly heterogeneous group that can be divided into two main classes depending on the mode of transposition (Bourque et al., 2018; Quesneville, 2020). Class I transposons (retrotransposons) propagate via RNA intermediates, and their “copy-and-paste” transposition mechanism results in the doubling of element copies with each transposition cycle (Mhiri et al., 2022). As a result, retrotransposons with long terminal repeats (LTRs) can account for up to 80– 90 % of the total transposon content and are the most abundant in plant genomes. Class II transposons (DNA transposons) are predominantly mobilized by a “cut-and-paste” mechanism, which usually does not result in an increase in transposon copy number. However, transposons such as Helitrons and MITEs can achieve high copy numbers in some genomes.

The distribution and accumulation of transposons is shaped by genetic drift and selection (Mhiri et al., 2022). New insertions usually have deleterious effects and are removed from the population. However, transposons can also undergo positive selection and promote adaptation (Niu et al., 2019). Transposons peak during periods of stress, allowing genomes to rearrange and rapidly diversify (Schrader and Schmitz, 2019).

Although associations of transposons with numerous agronomic traits are well documented (Catlin, Josephs, 2022), their contribution to crop phenotypic variability remains poorly understood (Akakpo et al., 2020; Alioto et al., 2020). Here, we investigated the chickpea mobilome composition by analyzing transposon insertions in 190 genomes of chickpea accessions from the VIR collection.

## Materials and methods

**Fig. 1.**
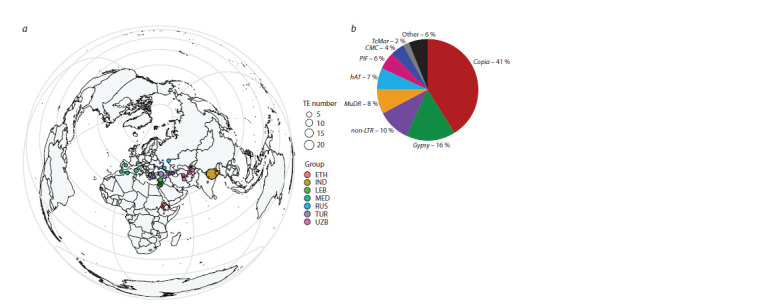
Collection sites of landraces (a); proportion of insertion sites for the most represented transposon superfamilies (b).

Plant material. 190 chickpea accessions from the collection of N.I. Vavilov All-Russian Institute of Plant Genetic Resources (VIR, St. Petersburg, Russia) were used in this work. Of these, 22 accessions were elite varieties, and the remaining accessions were landraces collected by N.I. Vavilov during his expeditions in the 1920–1930s. Based on the geographical proximity of the collection sites, landraces were divided into seven groups: accessions collected in the Mediterranean (MED), Lebanon (LEB), southern Russia (RUS), Turkey (TUR), Uzbekistan (UZB), India (IND) and Ethiopia (ETH) (Fig. 1a).

Bioclimatic variables. We used latitude and longitude coordinates for chickpea sample collection regions to obtain values of nineteen bioclimatic variables (Supplementary Table S1)1. Bioclimatic variables represent annual, seasonal and monthly averages and extremes of temperature and precipitation and are widely used in biogeographic analysis, climate change studies and ecological modelling. Data were downloaded from the WorldClim database version 1.4 (Hijmans et al., 2005), which contains information on climatic conditions recorded between 1960 and 1990. Values of the variables were extracted using the ‘raster’ package in R (https://rspatial.org/raster/) at a spatial resolution of 30 angular seconds, corresponding to approximately 1 square kilometer at the equator.


Supplementary Materials are available in the online version of the paper: https://vavilovj-icg.ru/download/pict-2025-29/appx3.xlsx


DNA sequencing and search for transposon insertions. The DNeasy Plant Mini Kit (Qiagen, Germantown, MD, USA) was used to isolate DNA from leaves. DNA samples were sequenced at the Beijing Genomics Institute (BGI, China) using the Illumina protocol, generating 150-bp paired-end reads. A total of 7,700 Gbytes of raw data were obtained, comprising about 26 billion reads with an average of 25-fold coverage or about 37 Gbytes per sample. Reads were processed and aligned to the chickpea genome reference assembly ASM33114v1 (Varshney et al., 2013) using the bwa-mem software with the default parameters (Li H., Durbin, 2009). The search for transposon insertion sites and assessment of their representation were performed using the PoPoolationTE2 program (Kofler et al., 2011, 2016). PoPoolationTE2 requires reads mapped to a reference genome with masked transposon sequences and a set of such sequences. The transposon sequences can be either consensus sequences of families (e. g. from RepBase), or sequences that have been masked in the reference genome, or both. When reads are aligned to such a modified genome, transposon insertions will result in groups of discordant paired ends, where one read maps to the reference genome, and the other, to the transposon sequence, while correctly aligned paired ends indicate the absence of an insertion. Based on the position of the matched ends of the paired fragments, a physical stack track (pile-up) is generated. The physical coverages of overlapping paired ends are summed, resulting in a physical coverage track, the height of which reflects the number of paired ends that overlap the given position. Transposon insertion signatures are determined using a sliding window method, by scanning for peaks in the physical coverage that confirm the presence of an insertion.

PoPoolationTE2 implements two fundamentally different analysis modes. In the separate mode, each sample/population is processed separately. This is similar to running the PoPoolationTE2 pipeline several times, for each bam file separately. In joint analysis (joint mode), the physical pileup tracks of different samples are combined and a joint pileup track is created. Transposon insertion signatures are identified on this joint pileup track. When identifying insertion signatures using the identifySignatures utility, a minimum average physical coverage parameter of three was used. Further, in the separate analysis, the signatures were filtered by the maximum frequency of other transposons in a given site and the maximum frequency of structural variants (rearrangements) in a given site. Both parameters were set equal to zero. In the joint analysis, filtering was performed only by the maximum frequency of other transposons in a given site, equal to 0.05. Validation of key insertions was performed using the Integrative Genomics Viewer program, which allows visualization of read alignment at the insertion site.

The search for hotspots of transposon insertions was performed by the PrimatR program (https://github.com/ daewoooo/primatR). The hotspotter function was used, which compares (within a 50 kb window) the density distributions of randomly scattered points, the number of which is equal to the number of transposons in the genome and the transposon location densities obtained from the experiment. The higher the value of transposon density in a given region, the more extreme it is for the distribution of “random” densities, and, therefore, the lower the p-level of significance. Hot spots were defined by p-values less than 1e–8.

Genetic data analysis. The population structure of the data was assessed using the ADMIXTURE v.1.3.0 program (Alexander et al., 2009). The Mann–Whitney–Wilcoxon test (Mann, Whitney, 1947) was used to compare groups.

Genome-wide association studies. Phenotyping of chickpea accessions was carried out at two VIR experimental stations, in Kuban and Astrakhan, as described earlier (Duk et al., 2024). 12 phenological and morphologyical traits were measured: plant height (PH), height of first pod (HFP), number of first order branches (NPB), number of second order branches (NSB), plant dry weight with pods (PWwP), pod weight per plant (PoW), pod number per plant (PoNP), 100 seeds weight (100SW), leaf size (LS), number of days from germination to flowering (DFst), flowering duration (DF), number of days from germination to full maturity (Dmat) (Table S2).

Phenotypic data from two experimental stations were quantile normalized. GWAS was performed using the FarmCPU, Blink, SUPER and MLMM programs of the GAPIT3 package for R with parameters MAF = 0.05 and FDR = 0.9. In addition, the IIIVmrMLM program in Single_env mode with parameters svpal = 0.01 (Li M. et al., 2022a, b) was also used for association studies. The IIIVmrMLM model was designed to address methodological shortcomings in detecting all types of interactions between alleles, genes, and environments, and to unbiasedly estimate their genetic effects. As a multilocus MLM model, IIIVmrMLM estimates the effects of all genes and the effects of all interactions simultaneously. However, IIIVmrMLM is less computationally complex, since the calculation of a large number of variance components has been replaced by the calculation of only three estimates. In addition, all effects in IIIVmrMLM are estimated within a single multilocus model using the Bayesian expectation-maximization algorithm, and all non-zero effects are further assessed using a likelihood ratio test for significant associations. All this actually guarantees accurate detection of insertion regions, unbiased estimation of their effects and makes IIIVmrMLM a good choice for detecting associations between markers, traits and the environment.

Information on the coordinates of candidate genes, containing markers in genes or in 1-kb flanking regions, was obtained from the GFF file version 1 Cicer_arietinum_GA_v1.0.gene. gff, and functional description of genes was obtained from the Pulse Crop Database (https://www.pulsedb.org/Analysis/ 1869759).

## Results

Composition of the chickpea mobilome

A total of 105 transposon families have been annotated in the chickpea reference genome (Varshney et al., 2013). To characterize novel transposon insertions in individual chickpea accessions, we analyzed whole-genome sequencing data from 190 samples, represented by 22 cultivated varieties and 168 landraces, which were divided into seven groups based on the sampling location (Fig. 1). A total of 42,324 new transposon insertion sites not represented in the reference genome were identified, with most sites being polymorphic and present in multiple accessions

Transposons of polymorphic insertion sites belong to 83 families and thus likely constitute the majority of the chickpea mobilome. Most insertions are due to mobilization of Copia (41 %) and Gypsy (16 %) retrotransposons (Fig. 1b, Supplementary Figure S1a)2 and 10 % account for insertions due to mobilization of non-LTR retrotransposons. Five superfamily groups – MuDR (8 %), PIF (6 %), hAT (7 %), CMC (4 %) and TcMar (2 %) – make the main contribution to the number of insertions caused by DNA transposons (Fig. S1b–f, Table S3).


Supplementary Materials are available in the online version of the paper: https://vavilov.elpub.ru/jour/manager/files/Suppl_StaninF_Engl_29_1.pdf


**Fig. 2.**
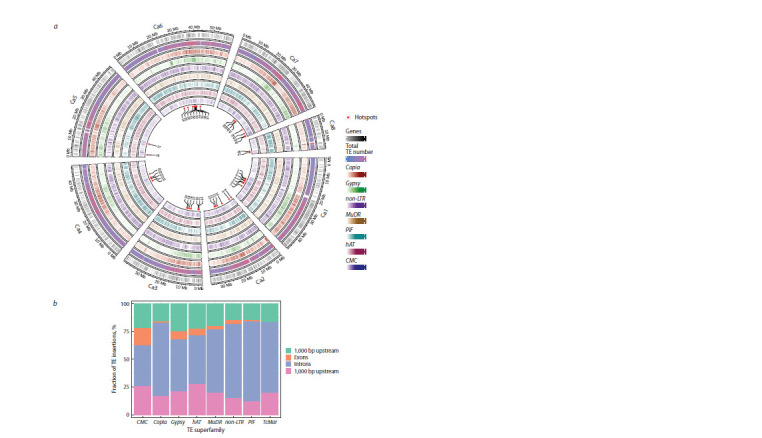
Distribution of transposon insertion sites of the most widely represented superfamilies and genes visualized using Circos software (a); distribution of transposon insertion sites relative to genes and their structural elements (b).

Polymorphic insertion sites are distributed unevenly along chromosomes (Fig. 2a, Table S4) and form 47 hotspots, with the lowest number of hotspots found in chromosomes 5 and 8. Sixteen hotspots contain exclusively retrotransposon insertions. Copia retrotransposon insertions were observed in all hotspots, and hAT, MuDR, PIF, and CMC DNA transposon insertions were observed in 60, 53, 23, and 47 % of the hotspots.

Chickpea mobilome landscape

From 15 to 22 % of the insertion sites of Copia elements, as well as elements of the MuDR, CMC, and hAT superfamilies, are located in genes or within 1 kb-flanking regions of genes (Fig. 2b, Table S5). In non-LTR retrotransposons, such insertions are about a third of the total number (35.33 %), and in DNA transposons of the TcMar and PIF superfamilies, they constitute half of the total number of such insertions (44.11 % and 57.93 %, respectively) (Table S5). The highest number of insertions in all transposon superfamilies occurs in introns (Fig. 2b), and the lowest, in exons. The largest excess of insertions in introns compared to exonic insertions was observed for the Copia and PIF superfamilies (42 times), the smallest, for Gypsy (6 times) and CMC (2.36 times). In TcMar, almost all gene-specific insertion sites fall into introns, and the flanking regions contain 30 times fewer insertions compared to introns. The greatest excess of transposon insertions in the flanking regions of genes compared to exons was observed for elements of the Copia, MuDR, and PIF superfamilies (10, 6, and 4 times, respectively) (Table S5).

Transposon insertion site polymorphism as a new source of phenotypic variability

To more systematically assess whether polymorphic insertion sites are a potentially important source of phenotypic variability, we used them as markers in the search for associations with agronomically important traits assessed at Astrakhan and Kuban VIR experiment stations in 2022 (Duk et al., 2024). GWAS was performed separately for each trait measured at each station using the GAPIT3 package for R and the IIIVmrMLM program in Single_env mode.

GAPIT3 package found 12 associations with three phenotypic traits: duration of flowering, number of days from germination to full maturity, height of the first pod, with one association between the DF trait and the insertion of the RTEBovB transposon at position Ca3_23488685 being found by two models (Table S6). The Ca3_1499163 and Ca6_24162635 insertion sites of the Copia and RC Helitron transposons associated with the height of the first pod are localized in the 5′-flanking regions of the Ca_19414 and Ca_11043 genes. These genes encode ribosomal protein S29 and late embryogenesis abundant protein, respectively.

**Fig. 3.**
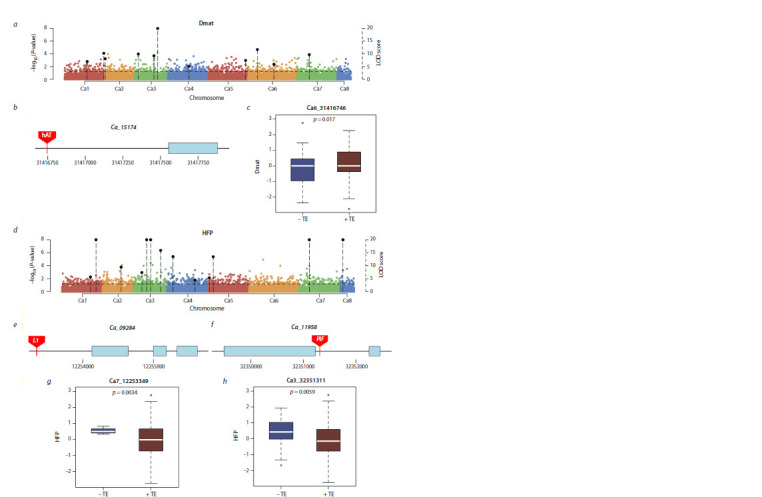
TE insertion sites as a source of phenotypic variability a – Manhattan plot showing associations of TE insertion sites with maturation time; b – Ca_15174 gene structure; c – maturation time of plants from accessions with and without TE insertion; d – Manhattan plot showing association of TE insertion sites with the height of the first pod; e, f – Ca_11958 (e) and Ca_09284 (f ) gene structure; g, h – height of the first pod in plants from accessions with and without TE insertion. See Figures S2–S4 for the results of transposon insertion validation

84 associations with Astrakhan station data and 114 associations with Kuban station data were found using the IIIVmrMLM program (Table S7). Three transposon insertion sites turned out to be polymorphic, in particular, Ca3_27767370, an insertion of the PIF-Harbinger transposon into the Ca_08130 gene (Table S8). This insertion was associated with pod weight per plant at the Kuban station and with days from germination to full maturity at the Astrakhan station. 47 transposon insertion sites were located in the genes or in their vicinity over a size of 1 kb. In most cases, however, these genes encoded proteins with unknown functions, and only 28 genes were functionally annotated (Table S9). An interesting example is the association of the hAT_Charlie transposon at position Ca6_31416746 with maturation time (Fig. 3a). This transposon is localized upstream of the Ca_15174 gene, encoding transcription factor from the CCHC(Zn) family (Fig. 3b). In alfalfa Medicago truncatula, deletion of the gene encoding such a transcription factor strongly reduces seed size, stem length, and internode length (Radkova et al., 2019). In Arabidopsis plants, transcription factors of the CCHC(Zn) family are involved in RNA metabolism, transcription elongation, polyadenylation, translation, pre-mRNA splicing, RNA export and degradation, microRNA and ribosomal RNA biogenesis, and post-transcriptional gene silencing. Transposon insertion extends pod maturation time (Aceituno-Valenzuela et al., 2020) (Fig. 3c).

The height of the first pod is an important trait for reducing harvest losses. The PIF-Harbinger transposon at position Ca3_32351311 is associated with this trait (Fig. 3d). It is localized in the Ca_11958 gene, which encodes the receptor for ethylene 2, a phytohormone that regulates plant growth and development (Fig. 3f ) (Binder, 2020). In rice, mutations in the gene encoding the ethylene 2 receptor have been shown to affect flowering time (Wuriyanghan et al., 2009). Another non-LTR transposon L1 at position Ca7_12253349, also associated with this trait, is located upstream of the Ca_09284 gene encoding chloroplast glucose-6-phosphate-1-dehydrogenase, which is involved in oxidative processes affecting germination, nitrogen metabolism, plant branching, and the response to abiotic stress (Jiang et al., 2022) (Fig. 3e). In both cases, plants with transposon insertion have a lower height of the first pod attachment, i. e. the transposon insertion has an unfavorable effect on the trait (Fig. 3g, h).

Comparison of the results of the association study using the GAPIT3 R package and the IIIVmrMLM program showed that four transposon insertions are detected by both programs (Table S10).

Polymorphism of transposon insertion sites in groups of chickpea landraces from different geographical locations

**Fig. 4.**
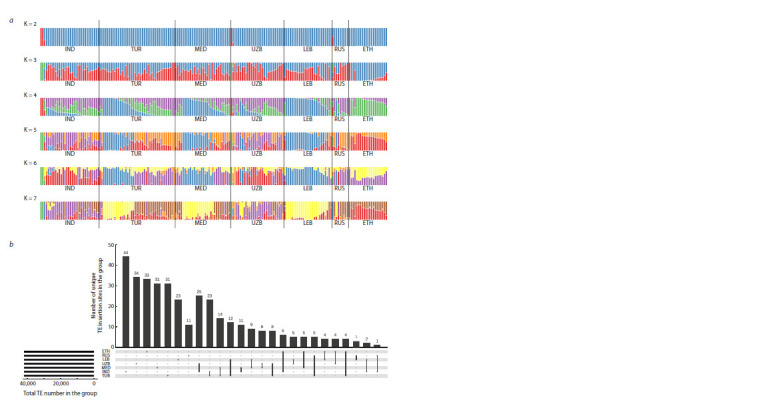
Transposon insertion sites as a source of diversification of samples from different geographical regions. а – population structure of landraces; b – Upset plot of transposon insertion sites. IND, MED, TUR, RUS, UZB, LEB, ETH – groups of accessions of different origin.

ADMIXTURE analysis of plink files made from data on transposon insertion sites showed that the most preferred number of populations was five, although the CV-error for four populations was actually the same (Fig. 4a). The population structure of accessions from different geographical groups (Fig. 1) differed. Accessions from Ethiopia (ETH) were the most contrasting compared to the other samples, the admixture patterns of Indian (IND) and Central Asian (UZB) samples were similar to each other and different from the admixture pattern of Turkish (TUR) and Mediterranean (MED) accessions. It can also be noted that Lebanese (LEB) and Ethiopian (ETH) accessions were the most homogeneous in terms of admixture patterns and differed most from each other

The number of polymorphic transposon insertion sites present in one group (unique sites) or in several, but not all groups of landraces, differed between groups (Fig. 4b, Table S11). Indian and Turkish accessions had the highest number of sites present in several groups, 650 and 705 sites, respectively. The Indian group also had the highest number of purely unique sites, namely, 44. The RUS group had the least number of unique insertion sites, which is likely due to the small number of samples in the group. The Ethiopian group stood out from all groups: it had the highest proportion (0.125) of unique sites among sites present in several groups. A more detailed analysis using the χ2 criterion revealed 514 insertion sites, the frequency of which in the groups differed from the theoretically expected frequency calculated under the assumption of no differences. Then, to examine the groups for enrichment in insertion sites, for each site with a non-random frequency of occurrence, we calculated two differences: between the maximum frequency value and the second highest frequency in the group, and between the minimum frequency value and the frequency second from the end (Table S12). This analysis confirmed that the Ethiopian population is enriched in transposon insertion sites that occur predominantly in this population, but also contains rare sites that occur frequently in other populations.

**Fig. 5.**
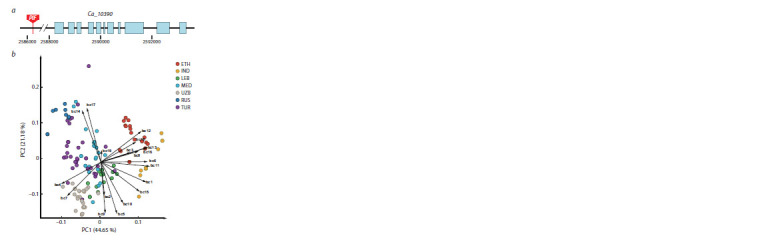
PIF-Harbinger transposon insertion in the Ethiopian group. a – structure of the Ca_10390 gene with transposon insertion; b – principal component plot of bioclimatic variables from the collection sites. The decoding of the bioclimatic variable labels is given in Table S1

It should be noted that the frequency of unique sites in groups, with rare exceptions, did not exceed 5 %, which indicates their relatively recent emergence. Only one PIFHarbinger transposon at position Ca6_2586225 in the Ethiopian group had a very high population frequency of 0.95. This transposon is 1,979 bp away from the Ca_10390 gene encoding the ROP-binding protein kinase RBK2 (Fig. 5a). RBK1/2 protein kinases phosphorylate small G-proteins of plant ROP and also interact with mitogen-activated protein kinase 1 (MPK1) from the auxin-responsive MPK cascade (Weiß et al., 2022). In addition, RBK1 is involved in Casparian strip formation and also plays a role in trichome branching, cytoskeleton stabilization and control of barley basal resistance to powdery mildew. Interestingly, the Ca6_2586225 position is located within a region of chromosome 6 about 100 kb long (2494265 to 2598131), which is virtually SNPfree in all C. arietinum samples. In addition to the Ca_10390 gene, this region contains nine other genes encoding proteins involved in hormone-mediated control of cell elongation, plant growth, transpiration, and formation of generative organs (Table S13).

## Discussion

Like all repetitive elements, transposons are characterized by extreme diversity. Each transposon family represents a continuum of more or less diverged copies, consisting of both autonomous and defective elements. This feature makes the identification and classification of transposons a challenging task, which has recently been progressively solved by highthroughput sequencing methods and the development of new programs. For example, the PoPoolationTE2 program used in this work (Kofler et al., 2016) implements an option for aggregating into one position TE insertion sites that differ by several nucleotides due to mapping inaccuracy (explained by sequence degeneracy). This allows to perform a comparative analysis of transposon insertions between different accessions.

By analyzing 190 chickpea genomes, we found 42,324 transposon insertion sites from 83 families and showed that most of the sites (70–80 %) are present in almost all accessions. The most abundant families were retrotransposons Copia (17,408 sites), Gypsy (6,813 sites), and non-LTR retrotransposons, represented mainly by L1 and RTE-BovB (4,245 sites) (Fig. 1b, Fig. S1). The percentage of DNA transposon insertion sites in chickpea accessions is significantly lower and the most common families are the family of Mu-like elements MuDR (8 %), as well as the PIF (6 %), hAT (7 %), CMC (4 %, represented mainly by CMC-EnSpm/CACTA) and TcMar (2 %) families. Copia family insertion sites are also prevalent in genomes of other plants (Domínguez et al., 2020; Cai et al., 2022). Our data are generally consistent with the results of the search for intact transposons in the chickpea reference genome, which also showed an excess of Copia family frequency over Gypsy and non-LTR frequencies, and the highest representation of the MuDR (Mu-like) family among DNA transposons (Mokhtar et al., 2021).

We found 47 transposon insertion hotspots, of which 16 contained exclusively retrotransposon insertions (Fig. 2a, Table S4). The non-random arrangement of transposon insertions of different families has also been demonstrated in other plant genomes (Sultana et al., 2017). For example, in tomato, Gypsy insertion sites are predominantly located in pericentromeric regions (Domínguez et al., 2020).

Transposon insertions can influence the expression of adjacent genes (Bourque et al., 2018); therefore, the analysis of their location relative to genes and their flanking regions is of interest. In chickpea, such regions were found to be enriched in transposon family insertion sites, which was particularly evident for non-LTR retrotransposon insertions as well as TcMar and PIF DNA transposons (Table S5). The enrichment of gene-specific regions and their flanking regions with transposon insertions has been demonstrated in many plants (Qiu et al., 2021; Zhao et al., 2022). In our data, transposon insertions were least frequently recorded in exons due to their deleterious effect and the action of negative selection. The highest number of insertions in all transposon superfamilies occurred in introns, which was particularly evident in the Copia, PIF, and TcMar superfamilies. In Copia and PIF, the excess of insertions into introns over exon insertions was 42-fold, and in TcMar, almost all gene-specific insertion sites fell within introns. The highest excess of transposon insertions in flanking regions of genes over exons was observed for elements of the Copia, MuDR, and PIF superfamilies (Table S5).

In joint mode analysis, transposon insertion signatures are reliably identified in individual accessions, which makes it possible to analyze the contribution of transposon insertion site polymorphism to phenotypic variation. We have shown that transposon insertion sites are an important factor diversifying phenotypes and can be successfully used in genome-wide association studies as markers replacing single nucleotide polymorphisms (Tables S6, S7). In this case, the IIIVmrMLM program finds significantly more associations between insertion sites and a trait than GAPIT3 R. This is partly explained by the fact that the strict threshold for the significance of associations implemented in GAPIT3 R excludes the possibility of identifying markers with small effects. The feasibility of using transposon insertion sites in genome-wide association studies has also been demonstrated in rice and tomato (Akakpo et al., 2020; Domínguez et al., 2020; Vourlaki et al., 2022; Yan et al., 2022).

Transposon insertion sites may have played a significant role in plant adaptation during evolution, since such changes can occur rapidly, which is critical for the organism to adapt to changing conditions (Niu et al., 2019; Schrader, Schmitz, 2019; Zhao et al., 2022; Kang et al., 2023). The primary domestication of chickpea occurred in the Fertile Crescent (modern Turkey), followed by secondary centers of diversification in India, Ethiopia, Central Asia and the Mediterranean (Igolkina et al., 2023). Due to the efforts of N.I. Vavilov, seeds of varieties from such centers are stored in the VIR collection, which makes it possible to study the polymorphism of transposon insertion sites in groups of accessions from different secondary diversification centers. It turned out that each group of accessions contained a large number of unique sites, but their frequency did not exceed 5 %, indicating their relatively recent emergence. Only one PIF-Harbinger transposon at position Ca6_2586225 in the Ethiopian group of samples had a very high population frequency of 0.95. It should be noted that, in terms of the admixture pattern, the population structure of Ethiopian varieties differed most significantly from other groups (Table S12). The transposon Ca6_2586225 is inserted into the 5ʹ-flanking region of the Ca_10390 gene encoding the ROP-binding protein kinase RBK2 (Fig. 5a), which is involved in the formation of the Casparian strip, i. e. in the regulation of the water balance of the plant (Weiß et al., 2022). As can be seen from the principal component analysis of bioclimatic variables from the collection sites (Fig. 5b), Ethiopian varieties are most dependent on the rainfall and humidity variables. This fact may be an indirect explanation for the spread of transposon Ca6_2586225 in the group, since such an insertion, with the determinant role of climatic variables associated with precipitation, may be adaptive and provide plants with a selective advantage.

Transposons are a major source of genomic mutations (Bourque et al., 2018). In the case of Ca6_2586225, the transposon insertion appears to have resulted in a beneficial change. More often, however, transposon insertions have a deleterious effect on a trait, as we see with transposon insertions at positions Ca7_12253349, Ca3_32351311 and Ca6_31416746 (Fig. 3b, e, f ).

## Conclusion

In this work, we performed a primary analysis of transposon insertion sites in a large number of chickpea accessions, represented mainly by landraces. We found high polymorphism of such sites, characterized the representation of different transposon superfamilies, and showed uneven distribution of insertion sites along chromosomes. We also showed that transposon insertion sites, which until recently were out of the field of population genomics, are an important factor diversifying phenotypes and ensuring plant adaptation to growing conditions. The data and results obtained in this study are a valuable resource that can be used as a starting point for a more in-depth analysis of the evolutionary dynamics of transposons in the chickpea genome, their contribution to adaptation to global climate change, and the breeding of new varieties.

## Conflict of interest

The authors declare no conflict of interest.
